# The patient’s anxiety before seeing a doctor and her/his hospital choice behavior in China

**DOI:** 10.1186/1471-2458-12-1121

**Published:** 2012-12-28

**Authors:** Liyang Tang

**Affiliations:** 1 Department of Economics, School of Economics and Management, Tsinghua University, Beijing 100084, China

## Abstract

**Background:**

The patient’s anxiety before seeing a doctor may influence her/his hospital choice behavior through various ways. In order to explore why high level hospitals were overused by patients and why low level hospitals were not fully used by patients in China, this study was set up to test whether and to what extent the patient’s anxiety before seeing a doctor influenced her/his hospital choice behavior in China.

**Methods:**

This study commissioned a large-scale 2009–2010 national resident household survey (N=4,853) in China, and in this survey the Self-Rating Anxiety Scale (SAS) was employed to help patients assess their anxiety before seeing a doctor. Specified ordered probit models were established to analyze the survey dataset.

**Results:**

When the patient had high level of anxiety before seeing a doctor, her/his level of anxiety could not only predict that she/he was more likely to choose the high level hospital, but also accurately predict which level of hospital she/he would choose; when the patient had low level of anxiety before seeing a doctor, her/his level of anxiety could only predict that she/he was more likely to choose the low level hospital, but it couldn’t clearly predict which level of hospital she/he would choose.

**Conclusion:**

The patient with high level of anxiety had the strong consistent bias when she/he chose a hospital (she/he always preferred the high level hospital), while the patient with low level of anxiety didn’t have such consistent bias.

## Background

What was the correlation between the patient’s anxiety before seeing a doctor and her/his hospital choice behavior?

The patient’s hospital choice behavior could be considered as the decision-making behavior in a general framework.

The decision-making that was an essential aspect of the cognitive functioning relied on the correct labeling, processing and control of emotional stimulus, since the patient’s anxiety exerted its influence on the cognitive functioning by altering the processing of environmental information in favor of the negative emotional stimulus, this situation further resulted in the attention, memory and interpretation biases towards such stimulus, then the patient’s anxiety that could be seen as the adaptive emotion adequately directed her/his response towards the possible threatening stimulus/situation to influence her/his decision-making behavior [1-6]. In fact, the patient’s anxiety and her/his decision-making behavior were proved to have a strong correlation with each other at the neural substrate level, several of the neural substrates played major roles in both anxiety and decision-making, and the patient’s anxiety may affect her/his decision-making behavior both at the level of emotional reactivity (related neural substrates involved the amygdala, the ventral striatum, and the orbitofrontal cortex) and at the level of cognitive control (related neural substrates involved the anterior cingulate cortex, the dorsolateral prefrontal cortex, and the dorsal striatum) [3,7-11].

In a more specific framework, the patient’s hospital choice behavior could be considered as the decision-making behavior under risk and uncertainty: choosing the appropriate type of hospital on the basis of both the specific disease and the patient’s current disease status was the process of asymmetric information between hospital and patient, the patient usually had little information on how to make the optimal hospital choice decision, and she/he could hardly detect the inappropriate hospital choice before seeing a doctor, but the inappropriate hospital choice was most likely to bring her/him both health losses and economic losses.

The psychological dimensions of risk and uncertainty not only contained a cognitive evaluation of the hazard, but also contained an affective evaluation of the hazard [12,13]. The patient’s emotion was not only found to significantly influence her/his tendency to take risk and uncertainty, but also found to significantly influence the impact of the framing of the problem on her/his risky and uncertain choice in a systematic and predictable way: the patient’s anxiety that was her/his negative emotion tended to make her/him more pessimistic about the future outcome of her/his hospital choice behavior, and this situation could ultimately lead to her/his risk aversion, in fact this phenomenon was proved true even when the patient’s anxiety resulted from the factors that were wholly unrelated to the issue whose risk she/he assessed [14-17]; the precise role of the patient’s anxiety also depended on the framing of the problem, if the problem involved risky and uncertain choices, the patient with high level of anxiety tended to act risk-averse when the problem was framed in terms of gains, while she/he tended to act risk-seeking when the same problem was instead framed in terms of losses [17], since the patient’s hospital choice problem was framed in terms of health gains and economic losses, and the health gains were usually considered more important than the economic losses by the patient with high level of anxiety, then she/he usually tended to act risk-averse when she/he made her/his hospital choice.

The patient’s anxiety before seeing a doctor could also be considered as the patient’s emotion in a general framework.

The patient’s emotional reactions to the stimuli were often more rapid and basic than her/his cognitive evaluations, and such immediate emotional responses provided the patient with the fast but crude assessments of the various types of hospitals that she/he faced, and this attribute of the patient’s emotions made it possible to take the rapid action to choose a hospital [18-22]. The patient’s rapid emotional reactions were also thought to serve as a mechanism that interrupted and redirected her/his cognitive processing towards the potential high-priority concerns (such as the imminent hospital choice behavior when she/he was sick) [13,23-26]. In fact the patient’s anxiety was postulated to play a critical role in her/his rational, risk-averse, and forward-looking decision-making, since the appropriate level of anxiety was argued to reflect the highest level of normal motivational control of the working memory that made the operation of working memory in the decision-making behavioral sequencing be continuously linked with the adaptive significance [27,28].

In contrast to the historical view that considered the patient’s emotions (particularly the patient’s anxiety) as the destructive influences on her/his decision-making, much of the new work highlighted the patient’s emotions (particularly the patient’s anxiety) as both the informational inputs into her/his decision-making and the negative consequences that resulted from the blocking of such inputs [13], and both the affect-as-information hypothesis and the risk-as-feeling hypothesis were the representative theories among these new studies [13]. Compared with other theories, the key feature of the affect-as-information hypothesis was that the patient’s affect had a direct effect on her/his decision-making rather than being mediated by the affect-congruent memory, and it correctly predicted that in the case where the patient’s feelings were experienced as the reactions to her/his imminent judgment/decision (such as her/his hospital choice), her/his feelings during the judgment process/the decision-making process affected her/his judgment/choice behavior [13,29,30]. The risk-as-feeling hypothesis postulated that the patient’s responses to her/his risky situation/decision-making resulted in part from her/his direct emotional influences: the patient evaluated the risk in choosing a hospital at a cognitive level based largely on the probability and desirability of associated consequences, and her/his feeling states that exerted reciprocal influences on her/his cognitive evaluations not only responded to the factors that did not enter into her/his cognitive evaluations of the risk (such as the immediacy of the risk in choosing a hospital), but also responded to both the probabilities and the outcome values of the risk in a way that was different from the way that her/his cognitive evaluations responded to both the probabilities and the outcome values of the same risk; the patient’s emotional reactions to the risk in choosing a hospital could diverge from her/his cognitive evaluations of the same risk due to their different determinants; then the patient’s hospital choice behavior that was the emotion-driven risk-related behavior was determined by the conflicting interplay between her/his cognitive evaluations and her/his feeling states (particularly her/his anxiety) [13].

Another explanation for the influence of the patient’s anxiety before seeing a doctor on her/his hospital choice behavior was that the patient’s anxiety influenced her/his choice behavior through influencing her/his perceived self-efficacy [31]. The patient’s perceived self-efficacy was concerned with her/his beliefs in the abilities to influence the events that affected her/his lives (such as choosing a hospital when she/he was sick), and the core belief of her/his self-efficacy was the foundation of human motivation, performance accomplishment, and emotional well-being [31-33]. In fact the patient relied partly on her/his physiological/emotional states and read her/his anxiety as one sign of personal vulnerability in judging her/his self-efficacy, and she/he had little incentive to undertake the hospital choice activity and persevere in the face of difficulty in her/his hospital choice decision-making unless she/he believed that she/he could produce the desired effects by her/his actions [31]. The patient’s self-efficacy belief influenced her/his selection of activities and environments when she/he needed to choose a hospital: the patient tended to avoid activities and situations that she/he believed exceeded her/his coping capabilities, but she/he readily undertook challenging activities and picked social environments that she/he judged herself/himself capable of handling when choosing a hospital [31].

In order to explore why high level hospitals were overused by patients and why low level hospitals were not fully used by patients in China, this study was set up to test whether and to what extent the patient’s anxiety before seeing a doctor influenced her/his hospital choice behavior in China. A large-scale 2009–2010 national resident household survey (N=4,853) in China was carried out in this study, and in this survey the Self-Rating Anxiety Scale (SAS) was employed to help patients assess their anxiety before seeing a doctor [34]. Specified ordered probit models were established to analyze the survey dataset.

## Methods

### Data

In order to collect the data on both the patient’s anxiety before seeing a doctor and the patient’s hospital choice behavior, this study collaborated with the National Bureau of Statistics of China to commission a large-scale 2009–2010 resident household survey in 17 provinces, autonomous regions, and municipalities directly under the central government.

In these 17 provinces, autonomous regions, and municipalities directly under the central government, under the support from the local Bureaus of Statistics, this survey adopted the two-stage probability proportional to size (PPS) systematic sampling technique to select a probability sample of 5,123 residents. The face-to-face interviews for this household survey were conducted by the professional survey teams from Tsinghua University and the local Bureaus of Statistics. The professional investigator usually first invited the resident to fill out the questionnaire on both her/his anxiety before seeing a doctor and her/his hospital choice behavior in the most recent medical experience. If the selected resident was away, refused to be interviewed, or failed to be interviewed after three attempts, no replacement was made. If the resident agreed to fill out the questionnaire, but she/he was unavailable, or disabled in a way that would impede her/him from filling out the questionnaire, another family member that knew her/him best served as the respondent, this family member was also asked to report her/his assessed values of the questions in the questionnaire to check bias. A total of 4,853 valid responses were generated, and the response rate of 94.73%.

The questionnaire for the 2009–2010 resident household survey consisted of three sections. The first section inquired about the resident’s certain personal characteristics (involving age, gender, marital status, education, income, employment status, job occupation, health status, medical insurance, reimbursement percentage of medical costs, severity of disease, and stage of disease in the most recent medical experience) that may influence her/his hospital choice behavior. The second section inquired about the resident’s anxiety before seeing a doctor in the most recent medical experience, here this study employed the SAS to help the resident assess 20 dimensions of her/his anxiety before seeing a doctor. Specifically, 20 dimensions of the self-rating anxiety included the dimension of anxiousness, the dimension of fear, the dimension of panic, the dimension of mental disintegration, the dimension of apprehension, the dimension of tremor, the dimension of body ache & pain, the dimension of easy fatigability & weakness, the dimension of restlessness, the dimension of palpitation, the dimension of dizziness, the dimension of faintness, the dimension of dyspnea, the dimension of paresthesia, the dimension of nausea & vomiting, the dimension of urinary frequency, the dimension of sweating, the dimension of facial flushing, the dimension of insomnia, and the dimension of nightmare. The corresponding questions for 20 dimensions of the self-rating anxiety were presented in Table 1. The third section inquired about the resident’s hospital choice behavior in the most recent medical experience.

**Table 1:** Dimensions of the self-rating anxiety and the corresponding questions from SAS

**Dimensions**	**Questions from self-rating anxiety scale (SAS)**
Anxiousness	I feel more nervous and anxious than usual.
Fear	I feel afraid for no reason at all.
Panic	I get upset easily or feel panicky.
Mental disintegration	I feel like I'm falling apart and going to pieces.
Apprehension	I feel that everything is all right and nothing bad will happen.
Tremor	My arms and legs shake and tremble.
Body ache & pain	I am bothered by headaches neck and back pain.
Easy fatigability & weakness	I feel weak and get tired easily.
Restlessness	I feel calm and can sit still easily.
Palpitation	I can feel my heart beating fast.
Dizziness	I am bothered by dizzy spells.
Faintness	I have fainting spells or feel like it.
Dyspnea	I can breathe in and out easily.
Paresthesia	I get feelings of numbness and tingling in my fingers and toes.
Nausea & vomiting	I am bothered by stomachache or indigestion.
Urinary frequency	I have to empty my bladder often.
Sweating	My hands are usually dry and warm.
Facial flushing	My face gets hot and blushes.
Insomnia	I fall asleep easily and get a good night's rest.
Nightmare	I have nightmares.

The personal characteristics of the study population were shown in Table 2. As the result of the stratified sampling design by the National Bureau of Statistics of China, the population distribution of each personal characteristic followed the natural distribution of residents in China. The use of the dataset was approved by the National Bureau of Statistics of China.

**Table 2:** Descriptive statistics of personal characteristics

**Dummy variables**	**Descriptions**	**Mean**	**Standard deviation**
Age dummy variables	Sample person is between 18–30 years old, 1=Yes, 0=Otherwise	0.255	0.436
	Sample person is between 31–45 years old, 1=Yes, 0=Otherwise	0.248	0.432
	Sample person is between 46–55 years old, 1=Yes, 0=Otherwise	0.249	0.432
Gender dummy variable	1=Male, 0=Female	0.504	0.500
Marital status dummy variables	1=Unmarried, 0=Otherwise	0.017	0.129
	1=Married, 0=Otherwise	0.910	0.286
	1=Divorced, 0=Otherwise	0.026	0.158
	1=Widowed, 0=Otherwise	0.045	0.207
Education dummy variables	1=Primary school or below, 0=Otherwise	0.094	0.292
	1=Junior high school, 0=Otherwise	0.268	0.443
	1=Senior high school, 0=Otherwise	0.260	0.439
	1=Secondary, 0=Otherwise	0.098	0.297
	1=College, 0=Otherwise	0.167	0.373
	1=University, 0=Otherwise	0.106	0.307
Income dummy variables	1=Income is less than ¥3390, 0=Otherwise	0.121	0.326
	1=Income is between ¥3390 and ¥5410, 0=Otherwise	0.124	0.330
	1=Income is between ¥5411 and ¥7420, 0=Otherwise	0.124	0.329
	1=Income is between ¥7421 and ¥9374, 0=Otherwise	0.126	0.332
	1=Income is between ¥9375 and ¥11700, 0=Otherwise	0.124	0.330
	1=Income is between ¥11701 and ¥15180, 0=Otherwise	0.127	0.333
	1=Income is between ¥15180 and ¥21860, 0=Otherwise	0.127	0.333
Employment status dummy variables	1=Employees of state-owned enterprises, 0=Otherwise	0.328	0.469
	1=Employees of various non-state-owned enterprises, 0=Otherwise	0.201	0.401
	1=Urban self-employed and private entrepreneurs, 0=Otherwise	0.068	0.252
	1=Homeworkers, 0=Otherwise	0.246	0.431
	1=Unemployed, to be distributed or other non-employed, 0=Otherwise	0.020	0.139
	1=Students, 0=Otherwise	0.042	0.202
	1=Reemployment of retired or retired personnel, 0=Otherwise	0.023	0.150
Job occupation dummy variables	1=Professional and technical personnel, 0=Otherwise	0.028	0.164
	1=Managers in government and government related enterprises, 0=Otherwise	0.141	0.348
	1=The clerk and manager, 0=Otherwise	0.214	0.410
	1=Commercial staff, 0=Otherwise	0.134	0.341
	1=Service staff, 0=Otherwise	0.002	0.050
	1=Farmers, animal husbandry and fishery workers, 0=Otherwise	0.103	0.304
	1=Production workers, transport workers and associated personnel, 0=Otherwise	0.001	0.032
Health status dummy variable	1=Average level or above average level, 0=Otherwise	0.842	0.364
Medical insurance dummy variables	1=Medical insurance for local urban workers, 0=Otherwise	0.553	0.497
	1=Medical insurance for local migrant workers, 0=Otherwise	0.003	0.056
	1=Self-financing medical insurance sponsored by the company or unit, 0=Otherwise	0.055	0.229
	1=Commercial medical insurance bought by employer, 0=Otherwise	0.005	0.072
	1=Privately purchased commercial medical insurance, 0=Otherwise	0.057	0.232
	1=Government funded health care reimbursement, 0=Otherwise	0.053	0.223
	1=The new rural cooperative medical insurance, 0=Otherwise	0.055	0.228
	1=Other medical insurance, 0=Otherwise	0.066	0.249
	1=No medical insurance, 0=Otherwise	0.148	0.355
Reimbursement percentage of medical costs dummy variables	1=Reimbursement percentage of medical costs is 100%, 0=Otherwise	0.071	0.256
	1=Reimbursement percentage of medical costs is between 70% and 99%, 0=Otherwise	0.175	0.380
	1=Reimbursement percentage of medical costs is between 40% and 69%, 0=Otherwise	0.148	0.355
	1=Reimbursement percentage of medical costs is between 20% and 39%, 0=Otherwise	0.060	0.238
	1=Reimbursement percentage of medical costs is between 1% and 19%, 0=Otherwise	0.032	0.175
Severity of disease dummy variables	1=Not serious, 0=Otherwise	0.395	0.489
	1=General, 0=Otherwise	0.448	0.497
	1=Serious, 0=Otherwise	0.112	0.315
Stage of disease dummy variables	1=Emergency and serious disease, 0=Otherwise	0.158	0.364
	1=Non-emergency disease at initial stage, 0=Otherwise	0.596	0.491
	1=Non-emergency disease at medium stage, 0=Otherwise	0.151	0.358
	1=Non-emergency stable disease at late stage, 0=Otherwise	0.095	0.293

### Measure of the patient's self-rating anxiety

The 4-item self-reporting measures that assessed 20 dimensions of the patient’s anxiety before seeing a doctor on a scale of 1 to 4 were employed, higher score reflected higher level of anxiety. Depending upon the suitability and usage of the SAS, the corresponding questions for 20 dimensions of the patient’s self-rating anxiety could be divided into two categories. The questions for the dimension of anxiousness, the dimension of fear, the dimension of panic, the dimension of mental disintegration, the dimension of tremor, the dimension of body ache & pain, the dimension of easy fatigability & weakness, the dimension of palpitation, the dimension of dizziness, the dimension of faintness, the dimension of paresthesia, the dimension of nausea & vomiting, the dimension of urinary frequency, the dimension of facial flushing, and the dimension of nightmare in the first category were worded symptomatically positive, for these questions, the option “Most or all of the time” was assigned score 4, the option “Good part of the time” was assigned score 3, the option “Some of the time” was assigned score 2, and the option “None or a little of the time” was assigned score 1. The questions for the dimension of apprehension, the dimension of restlessness, the dimension of dyspnea, the dimension of sweating, and the dimension of insomnia in the second category were worded symptomatically negative, for these questions, the option “None or a little of the time” was assigned score 4, the option “Some of the time” was assigned score 3, the option “Good part of the time” was assigned score 2, and the option “Most or all of the time” was assigned score 1. The mean value of the patient’s scores for 20 dimensions of her/his self-rating anxiety was her/his total average scoring of SAS.

### Measure of the patient's hospital choice behavior

According to the most recent “Governing rules for the management and classification of hospitals”, the public hospitals in China are classified as follows. The level 1 hospitals are “community hospitals or health clinics that provide direct prevention, treatment, health promotion, and rehabilitation services to participants of a defined community”. The level 2 hospitals are “area hospitals that provide comprehensive medical and other healthcare services to participants of multiple communities, which may, to a certain degree, also serve as teaching hospitals and research bases”. The level 3 hospitals are those that “provide high-quality, specialty medical and other healthcare services to participants in a minimum of several areas, and also serve as high-level teaching hospitals and conduct sophisticated research”. The community health centers serve as complementary health organizations to the three-level public hospital system, their functions are similar to the functions of the level 1 hospitals, but their sizes are smaller and their capacities are weaker than those of the level-1 hospitals, and compared with the level-1 hospitals, they mainly provide more junior direct prevention, treatment, health promotion, and rehabilitation services to participants of a defined community [35-37].

Then a 5-item self-reporting measure that assessed the patient’s hospital choice behavior on a scale of 0 to 4 was employed in this study: the option “Choose the level 3 hospital” was assigned score 4; the option “Choose the level 2 hospital” was assigned score 3; and the option “Choose the level 1 hospital” was assigned score 2; the option “Choose the community health center” was assigned score 1; and the option “Do not go to hospital or only go to pharmacy to buy the medicine for self-medication” was assigned score 0.

### Statistical methods

The ordered probit model is especially appropriate for this study, because the ordered probit discerns unequal differences between ordinal categories in the dependent variable-the patient’s hospital choice behavior [38-40]. For example, it doesn’t assume that the difference between choosing the community health center and choosing the level 1 hospital is the same as the difference between choosing the level 2 hospital and choosing the level 3 hospital. In fact the ordered probit in this study captures the qualitative differences among different hospital choice behaviors.

In the general form of the ordered probit model, the latent evaluation score y_i_ is a linear function of independent variables written as a vector x_i_, here i is the sample number, and y_i_=x_i_*b+ε_i_, where b is a vector of coefficients and ε_i_ is assumed to follow a standard normal distribution. For an ordered probit model with k cutoff points, define p_j_ (j=1,2,…,k) as the cutoff points of all y_i_, then y_i_≦p_1_, p_j_<y_i_≦p_j+1_ (j=1,2,…,k-1) or y_i_>p_k_. Following the notation, the general form of the ordered probit model is expressed as

(1) $$\mathrm{Prob}\left(y_{i}=y_{0}|x_{i}\right)=Ф\left(p_{1}-x_{i}*b\right)$$

(2) $$\mathrm{Prob}\left(y_{i}=y_{j}|x_{i}\right)=Ф\left(p_{j+1}-x_{i}*b\right)-Ф\left(p_{j}-x_{i}*b\right)\left(j=1,2,\ldots k-1\right)$$

(3) $$\mathrm{Prob}\left(y_{i}=y_{k}|x_{i}\right)=1-Ф\left(p_{k}-x_{i}*b\right)$$

where y_j_ (j=0,1,…k) is the discrete value of y_i_ and _Ф_ is the cumulative standard normal distribution function [37,41].

The marginal effect of x_i_ can be calculated according to this formula:

(4) $$\partial \mathrm{Prob}\left(y_{i}=y_{0}|x_{i}\right)/\partial x_{i}=-b*\varphi \left(p_{1}-x_{i}*b\right)$$

(5) $$\partial \mathrm{Prob}\left(y_{i}=y_{j}|x_{i}\right)/\partial x_{i}=-b*\left[\varphi \left(p_{j+1}-x_{i}*b\right)-\varphi \left(p_{j}-x_{i}*b\right)\right]\;\left(j=1,2,\ldots k-1\right)$$

(6) $$\partial \mathrm{Prob}\left(y_{i}=y_{k}|x_{i}\right)/\partial x_{i}=b*\varphi \left(p_{k}-x_{i}*b\right)$$

where φ is the standard normal density function, and based on (4), (5) and (6) the vector of coefficient b can be estimated [37,41].

On the basis of the general form of the ordered probit model, the following two specified ordered probit models were respectively estimated to test whether and to what extent 20 dimensions of the patient’s anxiety before seeing a doctor/the patient’s total average scoring of SAS influenced her/his hospital choice behavior:

(7) $$\mathrm{Model}\;1:y_{i}=\sum_{l}\beta_{l1}x_{\mathrm{li}}+\sum_{m}\beta_{m2}z_{\mathrm{mi}}+\varepsilon_{i}$$

(8) $$\mathrm{Model}\;2:y_{i}=\beta_{1}X_{i}+\sum_{m}\beta_{m2}z_{\mathrm{mi}}+\varepsilon_{i}$$

here i was the sample number; y_i_ was the patient’s hospital choice behavior; x_li_ (l=1,2,…,20) were respectively 20 dimensions of the patient’s anxiety before seeing a doctor, and X_i_ was the patient’s total average scoring of SAS; z_mi_ were control variables, since the patient’s hospital choice behavior may be influenced by her/his certain personal characteristics (involving age, gender, marital status, education, income, employment status, job occupation, health status, medical insurance, reimbursement percentage of medical costs, severity of disease, and stage of disease), they were all controlled as dummy variables in the specified ordered probit models; the error term ε_i_ was assumed to be distributed normal.

## Results

### Descriptive statistics of the patient's self-rating anxiety

The descriptive statistics of both 20 dimensions of the patient’s self-rating anxiety and the patient’s total average scoring of SAS were presented in Table 3. Averagely speaking, among 20 dimensions of the patient’s self-rating anxiety: patients had the highest level of anxiety in the dimension of easy fatigability & weakness, the dimension of palpitation, the dimension of anxiousness, the dimension of insomnia, the dimension of dizziness, the dimension of nightmare, the dimension of faintness, and the dimension of fear; and patients’ anxiety scores in the dimension of restlessness, the dimension of facial flushing, the dimension of panic, the dimension of apprehension, the dimension of sweating, and the dimension of urinary frequency were in the medium level; while patients had the lowest level of anxiety in the dimension of mental disintegration, the dimension of body ache & pain, the dimension of dyspnea, the dimension of tremor, the dimension of nausea & vomiting, and the dimension of paresthesia. The mean value of patients’ total average scorings of SAS was 2.614, and this value was also in the medium level.

**Table 3:** Descriptive statistics of the patient’s self-rating anxiety and hospital choice behavior

**Variables**	**Mean**	**Standard deviation**	**Min**	**Max**
Anxiousness	3.027	0.532	1	4
Fear	3.012	0.557	1	4
Panic	2.715	0.528	1	4
Mental disintegration	2.478	0.560	1	4
Apprehension	2.705	0.482	1	4
Tremor	1.844	0.518	1	4
Body ache & pain	2.229	0.572	1	4
Easy fatigability & weakness	3.042	0.515	1	4
Restlessness	2.890	0.470	1	4
Palpitation	3.035	0.515	1	4
Dizziness	3.018	0.521	1	4
Faintness	3.013	0.516	1	4
Dyspnea	2.103	0.484	1	4
Paresthesia	1.574	0.522	1	4
Nausea & vomiting	1.643	0.477	1	4
Urinary frequency	2.553	0.502	1	4
Sweating	2.583	0.518	1	4
Facial flushing	2.779	0.434	1	4
Insomnia	3.022	0.518	1	4
Nightmare	3.015	0.572	1	4
Total average scoring of SAS	2.614	0.116	1	4
The patient’s hospital choice behavior	3.153	0.893	0	4

### Descriptive statistics of the patient's hospital choice behavior

The descriptive statistics of the patient’s hospital choice behavior was also presented in Table 3. The mean value of the patient’s hospital choice behavior was 3.153, and this value was between the assigned score for the option “Choose the level 2 hospital” and the assigned score for the option “Choose the level 3 hospital”, or in other words, averagely speaking, patients were more likely to choose the high level hospitals (mainly referred to the level 2 hospital and the level 3 hospital) in the most recent medical experience, and this finding was consistent with China’s reality that high level hospitals were overused by patients, while the large number of medical resources in low level hospitals (mainly referred to the community health center and the level 1 hospital) were not fully used by patients.

### Regression analysis

The results of two specified ordered probit models were presented in Table 4. From the significance and size of the coefficient for each dimension of the patient’s self-rating anxiety in model 1/the significance and size of the coefficient for the patient’s total average scoring of SAS in model 2, the influence of the patient’s anxiety before seeing a doctor on her/his hospital choice behavior was revealed as follows.

**Table 4:** Regression results

	**(1)**	**(2)**
	**The patient’s hospital choice behavior**	
Total average scoring of SAS		0.827**
		(2.42)
Anxiousness	0.0612***	
	(3.74)	
Fear	0.0450***	
	(3.77)	
Panic	0.0241**	
	(2.09)	
Mental disintegration	0.0227**	
	(2.19)	
Apprehension	0.0234*	
	(1.95)	
Tremors	0.0748	
	(1.48)	
Body aches & pains	0.0203*	
	(1.69)	
Easy fatiguability & weakness	0.0632***	
	(3.81)	
Restlessness	0.0369**	
	(2.08)	
Palpitation	0.0701***	
	(3.36)	
Dizziness	0.0554***	
	(2.79)	
Faintness	0.0594***	
	(2.96)	
Dyspnea	0.0216*	
	(1.85)	
Paresthesias	0.0224	
	(0.47)	
Nausea & vomiting	0.0469*	
	(1.85)	
Urinary frequency	0.0343**	
	(1.96)	
Sweating	0.0414**	
	(2.20)	
Face flushing	0.0383**	
	(2.50)	
Insomnia	0.0484***	
	(3.94)	
Nightmares	0.0503***	
	(3.87)	
Age dummy variables	Yes	Yes
Gender dummy variable	Yes	Yes
Marital status dummy variables	Yes	Yes
Education dummy variables	Yes	Yes
Income dummy variables	Yes	Yes
Employment status dummy variables	Yes	Yes
Job occupation dummy variables	Yes	Yes
Health status dummy variable	Yes	Yes
Medical insurance dummy variables	Yes	Yes
Reimbursement percentage of medical costs dummy variables	Yes	Yes
Severity of disease dummy variables	Yes	Yes
Stage of the disease dummy variables	Yes	Yes
Cutoff point 1	−0.282	0.0960
	(−0.21)	(0.07)
Cutoff point 2	2.146	2.482*
	(1.57)	(1.88)
Cutoff point 3	2.921**	3.239**
	(2.14)	(2.45)
Cutoff point 4	3.125**	3.437***
	(2.29)	(2.60)
Number of observations	4853	4853
Log pseudo-likelihood	166.0	145.8

*t* statistics in parentheses, * *p* < 0.10, ** *p* < 0.05, *** *p* < 0.01.

From the regression result of model 1, almost all dimensions of the patient’s self-rating anxiety (except the dimension of tremor and the dimension of paresthesia) had significant positive influences on her/his hospital choice behavior. Among 20 dimensions of the patient’s self-rating anxiety: the patient’s anxiety in the dimension of anxiousness, the dimension of fear, the dimension of easy fatigability & weakness, the dimension of palpitation, the dimension of dizziness, the dimension of faintness, the dimension of insomnia, and the dimension of nightmare had the most significant and largest positive influences (p<0.01) on her/his hospital choice behavior; and the positive influences of the patient’s anxiety in the dimension of panic, the dimension of mental disintegration, the dimension of restlessness, the dimension of urinary frequency, the dimension of sweating, and the dimension of facial flushing on her/his hospital choice behavior were the second most significant and the second largest (p<0.05); while the patient’s anxiety in the dimension of apprehension, the dimension of body ache & pain, the dimension of dyspnea, and the dimension of nausea & vomiting had the least significant and smallest positive influences (p<0.1) on her/his hospital choice behavior; but the patient’s anxiety in the dimension of tremor or the dimension of paresthesia had no significant influence on her/his hospital choice behavior.

From the regression result of model 2, the patient’s total average scoring of SAS that can be considered as the comprehensive assessment of the patient’s anxiety before seeing a doctor had significant positive influence (p<0.05) on her/his hospital choice behavior. Through comparing the sizes of the coefficients between model 1 and model 2, the influence of the patient’s total average scoring of SAS was almost equal to the sum of the influences of all 20 dimensions of the patient’s self-rating anxiety.

From the values of cutoff point 1–4 in model 1, the assigned score for the option “Do not go to hospital or only go to pharmacy to buy the medicine for self-medication”, the assigned score for the option “Choose the community health center”, and the assigned score for the option “Choose the level 1 hospital” were between cutoff point 1 and cutoff point 2, and the assigned score for the option “Choose the level 2 hospital” was between cutoff point 3 and cutoff point 4, while the assigned score for the option “Choose the level 3 hospital” was above cutoff point 4. These cutoff point related results in model 1 revealed that 20 dimensions of the patient’s self-rating anxiety together with the patient’s certain personal characteristics could accurately predict which kind of high level hospital she/he would choose, but they could not clearly predict which kind of low level hospital she/he would choose.

From the values of cutoff point 1–4 in model 2, the assigned score for the option “Do not go to hospital or only go to pharmacy to buy the medicine for self-medication” was below cutoff point 1, both the assigned score for the option “Choose the community health center” and the assigned score for the option “Choose the level 1 hospital” were between cutoff point 1 and cutoff point 2, and the assigned score for the option “Choose the level 2 hospital” was between cutoff point 2 and cutoff point 3, while the assigned score for the option “Choose the level 3 hospital” was above cutoff point 4. These cutoff point related results in model 2 also revealed that the patient’s total average scoring of SAS together with the patient’s certain personal characteristics could accurately predict which kind of high level hospital she/he would choose, but they couldn’t clearly predict which kind of low level hospital she/he would choose.

## Discussion

### Main findings of this study

The main findings in the results section were summarized as follows: when the patient had high level of anxiety before seeing a doctor, her/his level of anxiety could not only predict that she/he was more likely to choose the high level hospital, but also accurately predict which level of hospital she/he would choose; when the patient had low level of anxiety before seeing a doctor, her/his level of anxiety could only predict that she/he was more likely to choose the low level hospital, but it couldn’t clearly predict which level of hospital she/he would choose.

This study also showed in detail that among 20 dimensions of the patient’s self-rating anxiety: when the patient had high level of anxiety in the dimension of anxiousness, the dimension of fear, the dimension of easy fatigability & weakness, the dimension of palpitation, the dimension of dizziness, the dimension of faintness, the dimension of insomnia, and the dimension of nightmare, her/his level of anxiety in these dimensions could best predict which level of hospital she/he would choose; when the patient had high level of anxiety in the dimension of panic, the dimension of mental disintegration, the dimension of restlessness, the dimension of urinary frequency, the dimension of sweating, and the dimension of facial flushing, her/his level of anxiety in these dimensions could second-best predict which level of hospital she/he would choose; when the patient had high level of anxiety in the dimension of apprehension, the dimension of body ache & pain, the dimension of dyspnea, and the dimension of nausea & vomiting, her/his level of anxiety in these dimensions could third-best predict which level of hospital she/he would choose; but the level of patient’s anxiety in the dimension of tremor and the dimension of paresthesia couldn’t be employed to predict which level of hospital she/he would choose. When the patient had low level of anxiety in all 20 dimensions of the self-rating anxiety, her/his level of anxiety in these dimensions could only predict that she/he was more likely to choose the low level hospital, but they couldn’t clearly predict which level of hospital she/he would choose.

### What is already known on this topic

On the basis of the extensive literature, the patient’s anxiety before seeing a doctor may influence her/his risky and uncertain decision-making behavior through various ways [1,13,17,31]. Since the patient’s hospital choice behavior could be considered as the representative risky and uncertain decision-making behavior, this study preliminarily derived that the patient’s anxiety before seeing a doctor may have significant influence on her/his hospital choice behavior. But there was no previous study that directly focused on the correlation between the patient’s anxiety before seeing a doctor and her/his hospital choice behavior.

### What this study adds

In a general framework, the patient’s hospital choice behavior could be simply considered as the decision-making behavior: the patient’s hospital choice decision-making that was a crucial aspect of her/his cognitive functioning relied on the correct labeling, processing and control of her/his anxiety stimulus; in fact the patient’s anxiety and decision-making also shared underlying neural substrates (such as the cortico-limbic pathways that included the amygdala, the striatum, and the medial/dorsolateral prefrontal cortices); the patient’s high level of anxiety was strongly associated with both the altered cognitive functioning and the attention, memory and interpretation biases towards the anxiety stimulus, and both of them further affected her/his hospital choice decision-making [1]. Due to these natural correlations between the patient’s anxiety and her/his decision-making behavior, the patient’s anxiety before seeing a doctor always had influence on her/his hospital choice behavior.

In a more specific framework, the patient’s hospital choice behavior could be considered as the decision-making behavior under risk and uncertainty: the patient’s anxiety state not only directly affected her/his risk and uncertainty assessment, but also conditioned the impact of the framing of the problem on her/his risk and uncertainty assessment in a systematic and predictable way, and the logic behind this was that the patient’s anxiety signaled a sense of danger and novelty that alerted her/him to stop, think, and adjust her/his choice behavior, in fact the influential model of affective intelligence held the view that the patient not only became more attentive to the external stimulus and the information-seeking, but also became more open to the attitude change when she/he was experiencing the negative emotions that generated anxiety; the patient’s anxiety also triggered her/his evaluation surveillance system that monitored the environment for the novel and threatening stimulus, interrupted the habitual routine, and engaged the thought [17,42-44]. And then both the direct impact and the indirect impact (via conditioning the impact of the framing of the problem) of the patient’s anxiety state on the risk and uncertainty assessment of her/his decision-making behavior further strengthened the influence of the patient’s anxiety before seeing a doctor on her/his hospital choice behavior.

The level of the patient’s anxiety was strongly and positively correlated with her/his risk aversion [45,46]. The patient’s anxiety on various types of judgments tended to favor her/his cautious and risk-averse decision-making [47,48]. The patient’s induced anxiety increased her/his preference for the low risk but low reward options [49]. To the extent that the patient’s risk aversion was the dominant response to her/his risky decision, her/his negative feelings (such as the anxiety) tended to dominate her/his positive feelings [13]. From the descriptive statistics of both 20 dimensions of the patient’s self-rating anxiety and the patient’s total average scoring of SAS, most patients in this study had high level of anxiety before seeing a doctor, then they were usually risk-averse in their hospital choice decision-making, and they usually preferred high level hospitals that were always more cautious and more risk-averse than choosing low level hospitals, even when the lower medical service expense/drug expense, the shorter waiting time before seeing a doctor, and the doctor’s more attention per patient in low level hospitals (took both the specific disease and the patient’s current disease status into account when choosing a hospital) could bring them positive feelings.

From the framework of the perceived self-efficacy, the patient’s high level of anxiety before seeing a doctor undermined and weakened her/his perceived self-efficacy on making the appropriate hospital choice to a large extent, and this situation ultimately reduced the patient’s incentive to undertake the hospital choice activity and persevere in the face of difficulty in her/his hospital choice decision-making [31-33]. Although the medical service expense/drug expense was higher, the waiting time before seeing a doctor was longer, and the doctor’s attention per patient was less in high level hospitals than those in low level hospitals, high level hospitals were always the safe choices for any kind of specific disease and any status of the patient’s current disease, and choosing a high level hospital didn’t need the patient’s careful consideration and comparison, then the patient with different sense of efficacy had different performance on her/his hospital choice behavior: the patient with high level of anxiety only had a low sense of efficacy on making the appropriate hospital choice, and this situation induced that she/he always preferred the high level hospital; while the patient with low level of anxiety had a strong sense of efficacy on making the appropriate hospital choice, and this situation induced that she/he usually preferred to make the appropriate hospital choice on the basis of her/his specific disease and current disease status.

The patient with high level of anxiety had the strong consistent bias when she/he chose a hospital (she/he always preferred the high level hospital), while the patient with low level of anxiety didn’t have such consistent bias. When the patient had high level of anxiety before seeing a doctor, both 20 dimensions of her/his self-rating anxiety and her/his total average scoring of SAS could accurately predict which level of hospital she/he would choose; when the patient had low level of anxiety before seeing a doctor, either 20 dimensions of her/his self-rating anxiety or her/his total average scoring of SAS couldn’t clearly predict which level of hospital she/he would choose.

The patient’s emotional reactions to a risky and uncertain situation often diverged from her/his cognitive evaluations of the same situation, and her/his emotional reactions not only often exerted a dominating influence on her/his hospital choice behavior, but also frequently produced the hospital choice behavior that did not appear to be adaptive [50]. For example, the patients that suffered from often-debilitating anxiety-related disorders were typically well aware that there was little or nothing to fear in the situation that they found so difficult, and the divergence of their emotional responses from their cognitive evaluations of risks and uncertainties, as well as the potency of their emotional responses in influencing their hospital choice behaviors were evident in them [27]. In fact the following vicious circle was getting more and more serious in China: the patients became more anxious before seeing a doctor, and then they were more risk-averse in their hospital choice decision-making, and this situation induced that they were more likely to choose the high level hospitals, while both the quality of medical service and the doctor’s medical service related competency in the low level hospitals couldn’t be promoted due to lack of medical practice that resulted from lack of patients, and this situation in turn increased the risks and uncertainties in choosing the low level hospitals, ultimately all the above situations increased the patients’ level of anxiety in making the optimal hospital choice before seeing a doctor.

### Limitations of this study

Several limitations should be noted. First, the response rate of the 2009–2010 national resident household survey was 94.73%, then the collected sample slightly deviated from the stratified sampling design, and this situation may have slight influence on the sizes of the coefficients in the regression results. Second, there may be other potential influencing factors for the patient’s hospital choice behavior that were not contained or controlled in this study, and this situation may influence the relative sizes of certain coefficients in the regression results. Third, the findings in this study were drawn on the basis of the sample in China, so this study could only interpret that the patient with high level of anxiety in China had the strong consistent bias when she/he chose a hospital (she/he always preferred the high level hospital), while the patient with low level of anxiety in China didn’t have such consistent bias, but it wasn’t clear whether these findings were correct only in China or in all countries with similar health delivery systems.

## Conclusion

This study found that the patient with high level of anxiety had the strong consistent bias when she/he chose a hospital (she/he always preferred the high level hospital), while the patient with low level of anxiety didn’t have such consistent bias. These findings were the valuable references for understanding why high level hospitals were overused by patients and why low level hospitals were not fully used by patients in China. From the aspect of health psychology, these findings also provided the useful suggestions for solving this problem in China’s future health delivery system reform.

But this study didn’t make it clear that whether the above findings were correct only in China or in all countries with similar health delivery systems. Since the patient’s anxiety before seeing a doctor influenced her/his hospital choice behavior through various ways, the author guessed that the above findings were correct in all countries with similar health delivery systems, and this conjecture could be the future research direction of this type of investigation in all countries with similar health delivery systems.

## Competing interest

The author declares that he has no competing interest.

## Authors’ contribution

LT designed the study, performed the statistical analysis, interpreted the results and drafted the manuscript.

## Pre-publication history

The pre-publication history for this paper can be accessed here:

http://www.biomedcentral.com/1471-2458/12/1121/prepub
